# Precision Psychobiotics for Gut–Brain Axis Health: Advancing the Discovery Pipelines to Deliver Mechanistic Pathways and Proven Health Efficacy

**DOI:** 10.1111/1751-7915.70079

**Published:** 2025-01-15

**Authors:** Rebecca F. Slykerman, Naomi Davies, Klara Vlckova, Kenneth J. O'Riordan, Shalome A. Bassett, James Dekker, Harriët Schellekens, Niall P. Hyland, Gerard Clarke, Elaine Patterson

**Affiliations:** ^1^ Department of Psychological Medicine University of Auckland Auckland New Zealand; ^2^ Fonterra Microbiome Research Centre University College Cork Cork Ireland; ^3^ APC Microbiome Ireland University College Cork Cork Ireland; ^4^ Fonterra Research and Development Centre Palmerston North New Zealand; ^5^ Riddet Institute Massey University Palmerston North New Zealand; ^6^ Department of Anatomy and Neuroscience University College Cork Cork Ireland; ^7^ Department of Physiology University College Cork Cork Ireland; ^8^ Department of Psychiatry and Neurobehavioural Science University College Cork Cork Ireland

**Keywords:** behaviour, GPCR, gut‐brain axis, microbiome, probiotic, psychobiotic, short chain fatty acids, tryptophan

## Abstract

Advancing microbiome–gut–brain axis science requires systematic, rational and translational approaches to bridge the critical knowledge gaps currently preventing full exploitation of the gut microbiome as a tractable therapeutic target for gastrointestinal, mental and brain health. Current research is still marked by many open questions that undermine widespread application to humans. For example, the lack of mechanistic understanding of probiotic effects means it remains unclear why even apparently closely related strains exhibit different effects in vivo. For the therapeutic application of live microbial psychobiotics, consensus on their application as adjunct treatments to conventional neuromodulators, use in unmedicated populations or in at‐risk cohorts with sub‐clinical symptomatology is warranted. This missing information on both sides of the therapeutic equation when treating central nervous system (CNS) conditions makes psychobiotic research challenging, especially when compared to other pharmaceutical or functional food approaches. Expediting the transition from positive preclinical data to proven benefits in humans includes interpreting the promises and pitfalls of animal behavioural assays, as well as navigating mechanism‐informed decision making to select the right microbe(s) for the job. In this review, we consider how these decisions can be supported in light of information accrued from a range of clinical studies across healthy, at‐risk and pathological study populations, where specific strains have been evaluated in the context of gastrointestinal physiology, brain function and behaviour. Examples of successful, partial and unsuccessful translation from bench to bedside are considered. We also discuss the developments in in silico analyses that have enhanced our understanding of the gut microbiome and that have moved research towards pinpointing the host–microbe interactions most important for optimal gut–brain axis function. Combining this information with knowledge from functional assays across in vitro and ex vivo domains and incorporating model organisms can prime the discovery pipelines with the most promising and rationally selected psychobiotic candidates.

## Graphical abstract

Schematic of a psychobiotic evaluation process that could be followed to gain information about the mechanism(s) of action from in silico, in vitro and ex vivo approaches to improve success rates in the discovery pipeline.

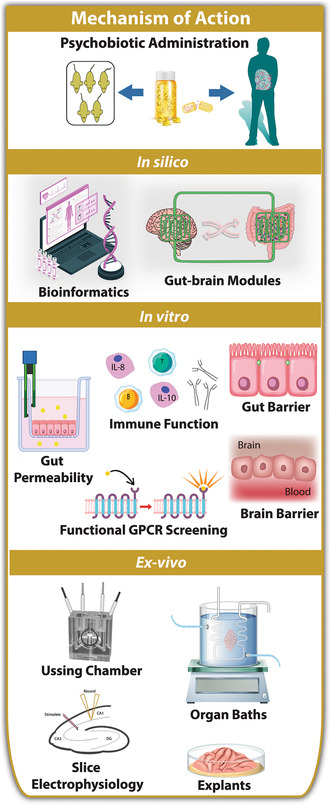

## 1 Introduction

Microbiome modulation represents an important example of where health benefits can potentially be accrued by deploying this approach to overcome current grand challenges. Mental health, in particular, is an important research priority area under the banner of ‘Good Health and Wellbeing’ in the Sustainable Development Goals (Statistics 2024) that could benefit from exploiting microbial biotechnology to deliver novel prophylactic and therapeutic options. The World Health Organization defines mental health as a state of mental wellbeing that allows people to cope with stress, reach their potential and contribute to their community and society (World Health Organization 2022). This definition outlines a concept of mental health that goes well beyond the simple absence of a mental disorder. Wellbeing is a similar concept that refers to a physical, mental, social and environmental status that gives rise to optimal psychological functioning and experience (Kiefer 2008). It is also well recognised that gut health and mental health are intricately linked and often co‐dependent, a relationship now understood to exist within the context of microbial inputs from the gastrointestinal tract (Skonieczna‐Zydecka et al. 2018).

**FIGURE 1:**
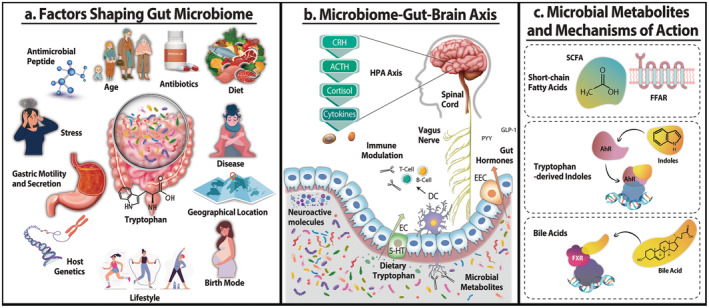
(a) A number of factors are known shape the composition and function of the gut microbiome as it processes building blocks like tryptophan to generate bioactives relevant at both levels of the gut–brain axis. (b) The microbiome–gut–brain axis has a number of key pillars including the immune and endocrine system, the enteric nervous system and the vagus nerve. Bacteria in the gut microbiota also act like mini‐factories, churning out microbial metabolites like SCFAs. (c) There are defined receptor‐based mechanisms of action for microbial metabolites such as FFAR for SCFAs, AhR for indoles and FXR for bile acids.

Long before the role of the gut microbiome was on the radar as a key regulator of brain function and behaviour, the framework and importance of the gut–brain axis was well appreciated (Cryan et al. 2019). This bidirectional communication network (Figure 1) brings together several key physiological systems including the immune system, hypothalamic–pituitary–adrenal (HPA) axis, the enteric nervous system and neural routes of communication, such as the vagus nerve (Wilmes et al. 2021). Together, these pathways facilitate control over fundamental aspects of gastrointestinal physiology, such as motility, sensation and secretion (Hyland and Cryan 2016), and more complex behaviours related to mood, emotion and cognition (Kelly et al. 2019). The gut microbiome, positioned at the core of this signalling framework, offers potential for a broad scope of influence on host physiology and behaviour, thus becoming an integral component of the now more appreciated microbiome–gut–brain axis.

In 2013, the term psychobiotic was introduced as ‘a live microorganism that, when ingested in adequate amounts, produces a health benefit in patients suffering from psychiatric illness’ (Dinan, Stanton, and Cryan 2013). This definition was subsequently expanded to include other means of influencing the gut microbiome to produce gut–brain health benefits in a wider variety of demographics, with probiotics now representing a subset within the psychobiotic class (Sarkar et al. 2016). Prebiotics are also an important subset of psychobiotic that warrant attention but are beyond the scope of this review. By signalling through the gut, probiotics with mental health benefits may reduce symptoms of depression and/or anxiety, or improve mood and mental wellbeing (Sarkar et al. 2016; Cryan et al. 2019). While the exact nature of the pathway or pathways that underlie this signalling remain to be fully elucidated, there is evidence that psychobiotics act by modulating neurotransmitters such as serotonin and γ‐aminobutyric acid (GABA), reducing proinflammatory cytokines or acting on the HPA axis to modulate the stress response and stress perception (Misera et al. 2021; Ross et al. 2024). Host–microbe interactions along the gut–brain axis may also be facilitated by a variety of other factors including microbial metabolites (Connell et al. 2022), microbial components such as peptidoglycans (Tosoni, Conti, and Diaz Heijtz 2019) or other bacterial mediators delivered via micro‐vesicles (Dalziel, Spencer, and Young 2021). Important examples of key microbial metabolites include short chain fatty acids (SCFAs) (O'Riordan et al. 2022), indoles derived from dietary tryptophan (Roager and Licht 2018; Gheorghe et al. 2019), bile acids (Long, Gahan, and Joyce 2017) and p‐cresol (Connell et al. 2022; Laudani et al. 2023). These microbial mediators can interact directly with host receptors (Figure 1) such as bacteria‐derived tryptamine which can act as a ligand for the gut epithelium‐expressed 5‐HT4 G‐protein coupled receptor (GPCR) to regulate colonic secretion and gut motility (Bhattarai et al. 2018; Cryan et al. 2018). Other host receptors of importance include the aryl hydrocarbon receptor (AhR), a ligand‐activated transcription factor and key sensor receptor for microbial metabolites (Barroso et al. 2021). Alternatively, microbially produced metabolites can indirectly regulate host metabolic pathways, an important example being the action of SCFAs on host tryptophan processing in the gut by enterochromaffin cells (Reigstad et al. 2015; Yano et al. 2015).

To date, an expanding array of potential therapeutic targets continues to accrue through which gut–brain axis function, gut physiology and behaviour can be modulated. In this review, we will take a closer look at the evidence for psychobiotics to influence human mental health and summarise some of the learnings gathered from preclinical models to unravel a route towards future successful psychobiotic discovery and clinical translation.

## 2 Psychobiotic Discovery From Bench to Bedside: Success and Failure in Translation

Given the complexity of the human brain, the rising global prevalence of mental illness, and the multi‐faceted and range of psychiatric disorders in humans, it is not surprising that animal models have been employed to help unravel underlying mechanisms, isolate pathways and define specific pathophysiological and neurobiological features (Slattery and Cryan 2014). This includes the preclinical screening and characterisation of compounds promoting mental wellbeing in humans. However, like many research applications, the use of animal models, widely used in this particular research field, faces challenges around translation into humans. These include how well an animal model recreates all the salient features of human disorders, and how well cause‐and‐effect relationships are preserved from animal models into humans. This is a particular challenge for complex heterogeneous psychiatric disorders where clinical diagnosis is based on clusters of symptoms that are often different between individuals within the same diagnostic category and that cannot always be simultaneously recapitulated in animal models or evaluated by the available behavioural assays (Gururajan et al. 2019). Thus, no one animal model is perfect, but each is useful for a specified purpose and the collective information from multiple approaches is needed to collate the necessary information across symptoms and diagnostic categories (Geyer and Markou 1995).

One approach to assess the usefulness of animal models is to consider three aspects of ‘validity’ (Willner 1984): *predictive validity* relates to whether the animal model predicts the performance of treatments to that condition; *face validity* refers to how closely the manifestations of the psychiatric disorder in the animal model relates to those in humans; and *construct validity* refers to similarities in mechanisms between humans and the animal model. The difficulty in meeting all of these criteria for psychiatric disorders where the pathophysiology and aetiology are often unknown has resulted in a prioritisation of predictive validity as the most relevant condition to meet (Geyer and Markou 1995).

**FIGURE 2:**
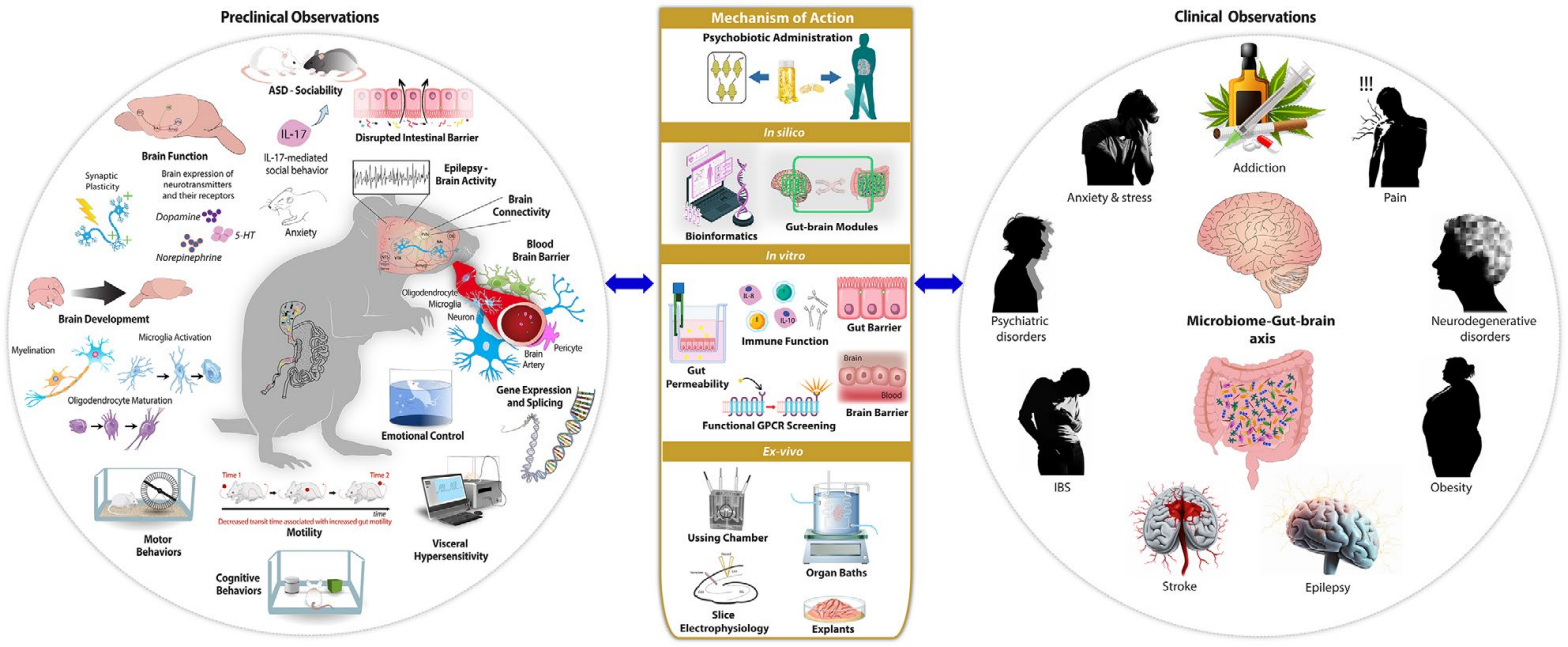
Our increased understanding of the role of the gut microbiota in brain function and behaviour incorporates observations from both clinical and preclinical studies. There are thus an expanding range of therapeutic targets for psychobiotics. The benefits of psychobiotic administration have been demonstrated in both animals and humans but information about the mechanism(s) of action has lagged behind these important observations. Such information can be gained from in silico, in vitro and ex vivo approaches to improve success rates in the psychobiotic discovery pipeline.

While a rapidly expanding body of research supports psychobiotic discovery (Figure 2), results of human trials remain diverse in their design and conclusions and the pathways to translation are not always straightforward. Initial translational attempts identified promising signals using preclinical screening assays, focusing translational efforts on often small groups of healthy adults. Although the results of these studies can be as diverse as the trial designs, there is a good indication that probiotics may indeed play a role in human mental health.

### 2.1 Microbial Interventions for Mental Wellbeing in Healthy Adults

One of the first examples of a positive translation of psychobiotic discovery from bench to bedside is that of *Bifidobacterium longum* 1714. Early preclinical studies demonstrated that this strain could reduce stress and anxiety‐like behaviour and depression‐related behaviour in innately anxious mice (Savignac et al. 2014). Some benefits to cognitive function were also described in a follow‐up study using the same mouse model (Savignac et al. 2015). In healthy adult men, *B. longum* 1714 reduced cortisol output in response to stress and attenuated the subjective anxiety response to the same experimental stressor after 4 weeks of intervention (Allen et al. 2016). Improvements in visuospatial memory performance and an electroencephalogram profile consistent with improved memory were also revealed (Allen et al. 2016). Further studies thereafter continued to demonstrate the psychobiotic effects of this strain. Wang et al. (2019) showed that *B. longum* 1714 modulated both resting state neural activity and neural responses to social stress, indicating a direct effect on brain processes linked to emotion. Moloney et al. (2021) demonstrated benefits to overall sleep quality and duration of sleep during exam stress. More recently, this beneficial impact on sleep quality was expanded to include improvements in daytime dysfunction due to sleepiness coupled with improved energy/vitality in a group of otherwise healthy individuals who exhibited impaired sleep quality (Patterson et al. 2024).

Stenman et al. (2020) examined 10 candidate probiotic strains in a preclinical behavioural test battery and reported efficacy for four of those strains in terms of reduced immobility in the forced swim test (FST) in stressed mice. Interestingly, one of the leading strains identified by their study, *Lacticaseibacillus paracasei* Lpc‐37, later showed mixed results in human studies (Makela et al. 2023; Patterson et al. 2020). This is despite the consistent and repeatable improvement across two preclinical studies of stress‐associated depression and anxiety (Stenman et al. 2020). It should be noted that there were significant protocol differences between these human studies such as the duration of the intervention (10 vs. 5 weeks) and the primary outcomes (state anxiety vs. heart rate in response to acute stress) which makes it challenging to understand the mixed results in terms of the psychobiotic potential of *L. paracasei* Lpc‐37. Furthermore, in the ‘Sisu study’, the Trier Social Stress Test was used to induce an acute and physiological stress response in healthy adults at the end of the intervention (Patterson et al. 2020), while the ‘ChillEx study’ employed a naturalistic model based on chronic examination stress in healthy students (Makela et al. 2023). This example highlights that standardised approaches for the evaluation of behavioural outcomes and endpoints are needed across both preclinical and clinical domains.

*Lacticaseibacillus rhamnosus* HN001 (Nutiani HN001) has been shown to reduce postpartum depression and anxiety when given to pregnant women from the second trimester to 6 months postpartum (Slykerman et al. 2017). It should be noted that stress exposures during pregnancy *per se* were not the focus of the maternal characteristics assessed in the study participants, but reductions in symptoms were evident across the range of depression and anxiety scores indicating the benefit was not restricted to only women with high postnatal depression or anxiety symptomatology (Slykerman et al. 2017). This result was mirrored in a recent rat study that investigated the influence of *L. rhamnosus* HN001 with and without stress on behaviours in pregnant rats (Lonstein et al. 2024). Consistent with the clinical observations, *L. rhamnosus* HN001 reduced stress‐associated maternal behaviours in rats including postpartum anxiety‐related behaviours, which was supported by the findings of reduced norepinephrine, dopamine and serotonin in the prefrontal cortex (Lonstein et al. 2024). However, when combined with stress during pregnancy, *L. rhamnosus* HN001 reduced time to immobility in the FST. At first sight, this would suggest increased depressive‐like activity in opposition to the clinical findings. However, it could also be interpreted as a positive adaptive response to save energy during pregnancy and lactation, highlighting some of the caveats and caution needed around the interpretation of the data from these preclinical observations. More recently in a healthy adult population, *L. rhamnosus* HN001 showed non‐significant improvements in both self‐reported happiness and perceived stress, further demonstrating its psychobiotic effects (Al Kassaa and Fuad 2024).

The combination of *Lactobacillus helveticus* R0052 and *B. longum* R0175 has been investigated across several preclinical and clinical studies since well over a decade with some findings translated in psychobiotic discovery and others showing mixed results. In one of the principal preclinical studies, it was originally revealed that this two‐strain combination reduced anxiety‐like behaviour in rats after only 2 weeks of intervention (Messaoudi et al. 2011). Later, healthy but borderline clinically anxious and depressed adults who consumed the same two‐strain combination reported improvements in anxiety and depression symptoms after a 30‐day intervention (Messaoudi et al. 2011). In a more recent trial, the combination did not improve any outcomes of psychological health (Morales‐Torres et al. 2023). However, in those participants with high health behaviour scores (as assessed using four‐point Likert scales on seven domains of lifestyle [diet, physical activity, sleep behaviour, nature exposure, social contact, social media use and substance abuse]), the two‐strain combination in conjunction with health behaviours was the single best predictor of reduction in anxiety, improvement in emotion regulation (the ability to effectively manage emotional responses in contrast to emotional dysregulation which reflects a state of poor emotional control that can be outwardly observed in altered behaviour or emotional distress), and mindfulness (Morales‐Torres et al. 2023). Aspects of emotional regulation can be measured using self‐report of coping, observer report of distress or records of altered behaviour. We acknowledge that there are challenges in measuring mindfulness although there are some tools under consideration (Bostanov et al. 2018). While there is some evidence of promising translation, results generated from diverse human study protocols highlight the challenges that heterogeneous study design presents to interpreting the results overall (see Table 1 for summary).

**TABLE 1:** Summary of psychobiotic interventions for mental wellbeing in healthy adults.

Psychobiotic	Reference	Sample	Intervention	Measures	Findings
*Bifidobacterium longum* 1714	Savignac et al. (2014) 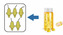	Innately anxious BALB/c mice (*n* = 22)	6 weeks Oral administration	Stress‐induced hyperthermia test, marble burying, elevated plus maze, open field, tail suspension test and forced swim test	Reduction in stress, anxiety‐like behaviours Reduction in depression‐like behaviours
	Savignac et al. (2015) 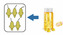	Adult male BALB/c mice (*n* = 48)	11 weeks Oral administration	Object recognition, barnes maze, fear conditioning, colorectal distension, plasma collection (for corticosteroid measurement)	Improvement in cognitive function
	Allen et al. (2016) 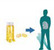	Healthy adult men (*n* = 20)	10 weeks total (4 weeks placebo, 4 weeks intervention, 2 weeks follow‐up) Repeated measures, placebo‐controlled trial Oral administration (mixed into milk)	Cohen Perceived Stress Scale (daily), Cambridge Neuropsychological Test Automated Battery, Emotional Stroop, EEG	Decreased cortisol output in stress response Attenuated anxiety Improved visuospatial memory and performance Improved memory (electroencephalogram)
	Wang et al. (2019) 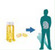	Healthy adults (*n* = 40)	4 weeks Randomised, double‐blind, placebo‐controlled trial	Cyberball game, 36‐item short‐form health survey, brain activity (magnetoencephalography)	Modulation of resting state neural activity and processes
	Moloney et al. (2021) 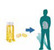	Healthy male students (*n* = 20)	8 weeks Randomised, placebo‐controlled, repeated measures, cross‐over intervention	Mini International Neuropsychiatric Interview (MINI) Battery of self‐report scales; Childhood Trauma Questionnaire‐Short Form (CTQ‐SF), Ten Item Personality Inventory (TIPI), Cambridge Behaviour Scale (CBS), Interpersonal Reactivity Index (IRI), Autism Quotient (AQ), State–Trait Anxiety Inventory (STAI) – trait part, and Ways of Coping Questionnaire (WAYS) Cambridge Neuropsychological Test Automated Battery	Beneficial impact on sleep quality and duration during exam stress
	Patterson et al. (2024) 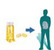	Healthy individuals with impaired sleep quality (*n* = 89)	8 weeks Randomised, double‐blind, placebo‐controlled, parallel‐groups, two‐arm (allocation ratio 1:1) clinical trial	Pittsburgh Sleep Quality Index (PSQI), Hospital Anxiety and Depression Scale (HADS), the Insomnia Severity Index (ISI), the Short Form Health Survey‐36 (SF‐36), and the Perceived Stress Scale (PSS)	Improvement in daytime dysfunction due to sleepiness coupled with improved energy/vitality
*Lacticaseibacillus paracasei* Lpc‐37	Stenman et al. (2020) 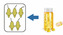	Five‐week‐old male Swiss mice	4 weeks Daily oral gavage	Elevated plus maze, open field, novel object recognition and forced swim test Corticosterone and adrenocorticotropic hormone	Decreased immobility in the forced swim test (FST)
	Patterson et al. (2020) 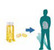	Healthy adults (*n* = 120)	5 weeks Randomised, double‐blind, placebo‐controlled, two‐arm (allocation ratio 1:1) and parallel‐groups clinical trial	Trier Social Stress Test was used to induce an acute and physiological stress response in healthy adults at the end of the intervention	Reduced perceived stress in the general population compared to placebo, particularly in females
	Makela et al. (2023) 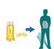	University students (*n* = 190)	10 weeks Triple‐blind, parallel, multicentre clinical trial	Naturalistic model based on chronic examination stress State Trait Anxiety Inventory (STAI‐state)	No effect on stress, mood or anxiety
*Lacticaseibacillus rhamnosus* HN001 (Nutiani HN001)	Slykerman et al. (2017) 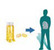	Pregnant women (*n* = 423)	A randomised, double‐blind, placebo‐controlled trial Second trimester to 6 months postpartum	Modified versions of the Edinburgh Postnatal Depression Scale (EPDS) State Trait Anxiety Inventory (STAI‐state)	Reduction in postpartum depression and anxiety
	Lonstein et al. (2024) 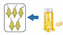	Pregnant adult female Long‐Evans rats	Oral administration (via cage water)	Maternal caregiving, elevated plus maze, saccharin preference, forced swim test, high performance liquid chromatography (brain tissue samples)	Decreased stress‐associated maternal behaviours including postpartum anxiety‐related behaviours Decreased norepinephrine, dopamine and serotonin in the prefrontal cortex
	Al Kassaa and Fuad (2024) 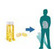	Healthy adults (*n* = 120)	28 days Double‐blind, placebo‐controlled trial	Perceived Stress Scale (PSS), Oxford Happiness Questionnaire (OHQ), Depression, anxiety and stress scale 21 item (DASS‐21)	Non‐significant improvements in both happiness and perceived stress
*Lactobacillus helveticus* R0052 and *Bifidobacterium longum* R0175	Messaoudi et al. (2011) 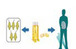	Rats: Male Wistar rats (*n* = 36) Humans: Healthy but borderline clinically anxious and depressed adults (*n* = 55)	Rats: 2 weeks intervention Gavage. Humans: 30‐days intervention Double‐blind, placebo‐controlled, randomised parallel group study Probio Stick	Rats: Conditioned defensive burying test (screening model for anti‐anxiety agents) Humans: Hopkins Symptom Checklist (HSCL‐90), the Hospital Anxiety and Depression Scale (HADS), the Perceived Stress Scale, the Coping Checklist (CCL) and 24 h urinary free cortisol (UFC)	Rats: Reduction in anxiety‐like behaviour Humans: Improvements in anxiety and depression symptoms

Morales‐Torres et al. (2023) 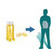	Healthy adults (*n* = 135)	12 weeks Randomised placebo‐controlled clinical trial	Ryff well‐being Scale (RYFF), Positive And Negative Affect Scales (PANAS), Satisfaction with Life Scale (SWLS), SF‐36 Health Survey, The State–Trait Anxiety Inventory (STAI), Difficulties in Emotion Regulation Scale (DERS), Multidimensional Assessment of Interoceptive Awareness (MAIA), Five Facet Mindfulness Questionnaire (FFMQ)	No improvement in any outcomes of psychological health
*Bifidobacterium longum* APC1472	Schellekens et al. (2021) 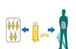	High‐fat diet (HFD)‐induced obese mice and healthy overweight/obese adults (*n* = 124)	Mice: 16 weeks Oral ingestion via drinking water Adults: 12 weeks Parallel‐controlled design Capsule	Body‐mass index, waist‐to‐hip ratio (W/H ratio) obesity‐associated plasma biomarkers	Positive translational effect on fasting blood glucose from a preclinical mouse model of obesity to a human intervention study in otherwise healthy overweight and obese individuals Improvements in fasting blood glucose but not body‐mass index or waist‐to‐hip ratio Reduced cortisol awakening responses in obese individuals only

Abbreviations: AQ, autism quotient; CBS, Cambridge behaviour scale; CTQ‐SF, childhood trauma questionnaire‐short form; DERS, SF‐36 Health Survey, difficulties in emotion regulation scale; EEG, electroencephalogram; EPDS, Edinburgh postnatal depression scale; FFMQ, five facet mindfulness questionnaire; FITC, fluorescein isothiocyanate; GSRS, gastrointestinal symptom rating scale; HADS, hospital anxiety and depression scale; HSCL‐90, Hopkins symptom checklist; IRI, interpersonal reactivity index; ISI, the insomnia severity index; LEIDS‐R, Leiden index of depression sensitivity‐revised; MAIA, multidimensional assessment of interoceptive awareness; MINI, mini international neuropsychiatric interview; PANAS, positive and negative affect scales; PSQI, Pittsburgh sleep quality index; PSS, the perceived stress scale; RYFF, Ryff well‐being scale; SF‐36, the short form health survey‐36; STAI, State–Trait anxiety inventory – trait part; SWLS, satisfaction with life scale; the CCL, coping checklist; TIPI, ten item personality inventory; UFC, 24 h urinary free cortisol; WAYS, ways of coping questionnaire.

*Note:* Green shading represents studies in humans, blue shading represents studies in rodents and orange shading represents papers reporting results from both human and rodent studies.

Partial successful translation from rodent to human was demonstrated for the anti‐obesity effects of *B. longum* APC1472 on the secondary outcomes of fasting blood glucose but not body‐mass index or waist‐to‐hip ratio (Schellekens et al. 2021). This strain also reduced the cortisol awakening response in obese individuals only, consistent with the reduction in stress‐induced corticosterone circulating levels in mice exhibiting high‐fat diet‐induced obesity.

Other examples of successful translation come from the use of microbial metabolites. Preclinical studies in mice illustrated the potential benefits of SCFAs with oral supplementation of a mixture of acetate, propionate and butyrate alleviating stress‐induced brain–gut axis alterations in reward‐seeking behaviour, responsiveness to an acute stressor and in vivo intestinal permeability (van de Wouw et al. 2018). Cecal acetate elevation was also associated with anxiolytic effects in mice (Kimura‐Todani et al. 2020). It was subsequently demonstrated in healthy human subjects that a mixture of colon delivered SCFAs blunted cortisol output to a psychosocial stress exposure (Dalile et al. 2020). Interestingly, this effect does not appear to be associated with butyrate alone as colonic administration of this SCFA did not modify the cortisol stress response to the same acute stressor (Dalile et al. 2024).

### 2.2 Psychobiotics in Adults With Chronic Stress and Psychiatric Disorders

Stress is a pervasive experience that can threaten mental health and wellbeing and is a major risk factor for neuropsychiatric and gastrointestinal disorders (Ju et al. 2020; O'Mahony et al. 2017; Kessler et al. 2010). Both acute and chronic stress impact the gut microbiota composition and intestinal permeability (Madison and Bailey 2024; Leigh et al. 2023). The gut microbiome in turn programs the HPA axis for appropriate responses to stress exposures (Sudo et al. 2004; Lyte et al. 2020), highlighting the reciprocal nature of what we now know to be an enduring relationship, implicating the gut microbiome as an important therapeutic node in the gut–brain axis for stress‐recovery and resilience (Foster, Rinaman, and Cryan 2017; Clarke et al. 2014). This explains why stress readouts are high on the agenda of studies using probiotic manipulations of the gut microbiome and why microbiome modulation could mitigate the effects of stress on the microbial environment, support physiological resilience to the experience of stress and counteract or prevent the effects on stress in psychiatric disorders (Madison and Bailey 2024). While a recent systematic literature review concluded that the evidence for psychobiotic efficacy in specific psychiatric disorders was mixed, there is encouraging evidence that psychobiotics could improve scores on measures of stress and anxiety in adults with moderate stress levels (Vasiliu 2023). Furthermore, systematic and meta‐analysis of randomised controlled trials of probiotic supplementation in people with diagnosed mood disorders conclude that there are moderate to high levels of evidence that probiotics can alleviate symptoms associated with common psychiatric disorders including depression and anxiety (El Dib et al. 2021; Musazadeh et al. 2023; Goh et al. 2019; Ribera et al. 2024). However, others conclude that the evidence supporting psychobiotics is inconsistent for depression, anxiety and stress (Le Morvan de Sequeira et al. 2022). This diversity in conclusions underscores the challenges in systematically evaluating psychobiotics given the heterogeneity in design and outcome measures analysed.

When used in conjunction with standard antidepressant therapy, psychobiotics are effective in improving the severity of depression (Nikolova et al. 2021; Firth et al. 2019; Eskandarzadeh et al. 2021). There is also promising evidence for psychobiotics as an adjunct therapy in the treatment of anxiety disorders, for example, in conjunction with selective serotonin re‐uptake inhibitors (SSRIs) such as Sertraline (Eskandarzadeh et al. 2021; Forth et al. 2023). Improved symptom reduction with adjunctive psychobiotic therapy suggests synergistic effects of psychobiotics and antidepressants (Nikolova et al. 2023). Studies point towards reduced inflammation as a relevant mechanism of action where therapeutic improvements in response to psychobiotics have been identified (Ribera et al. 2024). Evidence to suggest candidate psychobiotics as a standalone treatment for depression is less conclusive. A recent meta‐analysis indicated that psychobiotics alone are insufficient to reduce patient symptoms in major depressive disorder (MDD) (Nikolova et al. 2021).

Current evidence shows mixed results in the alleviation of anxiety with psychobiotic supplementation (Vasiliu 2023; Liu et al. 2018). A meta‐analysis of 12 randomised controlled trials found no significant difference in anxiety symptoms attributable to psychobiotic supplementation, urging caution in promotion of psychobiotics for anxiety until further double‐blind studies are conducted. Three of the 12 trials included in the meta‐analysis reported significant anxiety improvement with psychobiotics, suggesting strain‐specific effects are potentially obscured when combined into meta‐analyses. Attempting to identify commonalities between these three studies that explained their positive results, Liu et al. (2018) examined the health status of participants, sample size, strain of psychobiotic used, dose and duration of intervention, and outcome assessment tool, and found no significant predictors of positive randomised control findings.

In other psychiatric diagnoses including psychosis, clinical studies suggest that psychobiotics may increase tolerability of antipsychotics (Nikolova et al. 2023) but there is currently no evidence to suggest psychobiotics alleviate symptoms (Le Morvan de Sequeira et al. 2022; Nikolova et al. 2023). Of note is the side effect profile of antipsychotic treatment which includes weight gain and metabolic syndrome, features which may be attributable to microbiota remodelling (Davey et al. 2013, 2012). Thus, improving metabolic sequelae of antipsychotic treatment could therefore be a therapeutic target for psychobiotics.

Indeed, the emergent field of pharmacomicrobiomics could be of particular relevance in the context of psychobiotics. There are extensive reciprocal interactions between xenobiotics and bacteria with potential beneficial and harmful implications (Clarke et al. 2019). This is particularly notable in the context of the psychotropic drugs used to treat mental health conditions (Walsh et al. 2018). The baseline microbiota configuration of an individual is an important factor influencing treatment response to a psychobiotic, which are often ingested as adjuncts to conventional antidepressants, anxiolytics and antipsychotics. There are now numerous indications that these drugs have antimicrobial properties and remodel gut microbiota composition (Cussotto et al. 2019; Maier et al. 2018; Vich Vila et al. 2020), meaning that psychobiotics could encounter a very different gut microbiome depending on the interface between the treatment regimen and gut microbiome of each patient with as yet unclear implications for efficacy. This may also be important in the context of the side effects associated with psychotropic drugs, such as the weight gain associated with antipsychotic use in the treatment of schizophrenia, where animal studies have implicated the gut microbiota in the associated weight gain phenotype (Davey et al. 2012, 2013).

An equally important consideration is whether psychobiotics might impact the pharmacokinetic profile of co‐administered psychotropic drugs. For instance, there is recent evidence of a role for the gut microbiota in contributing to the bioavailability of olanzapine (Cussotto et al. 2021) and levodopa (van Kessel et al. 2019). There are also indirect pharmacokinetic consequences to consider since the expression of hepatic genes implicated in drug metabolism and drug transport are sensitive to modifications of the gut microbiome (Walsh, Gheorghe, et al. 2020). It is also important to consider how gut microbial enzymes are influenced by microbiota manipulations and what this means for efficacy and side effects. For example, gut microbial β‐glucuronidase enzymes can reactivate glucuronides in the gut with implications demonstrated for luminal serotonin levels if the function of this enzyme is modified (Simpson et al. 2024; Walsh, Olavarria‐Ramirez, et al. 2020).

Recent consideration has also been given to the paucity of studies investigating the possible interactions between microbes, putative psychobiotics and drugs, which could ultimately influence drug bioavailability, efficacy and toxicity (Purdel et al. 2023). Evidence from human studies demonstrates that several drugs can be influenced by particular taxa or probiotics including benzodiazepines (Purdel et al. 2023). For example, co‐administration of drugs used to manage Parkinson's disease along with *Bifidobacterium animalis* subsp. *lactis* Probio‐M8, significantly improved patients' disease scores (Sun et al. 2022). Moreover, this probiotic was associated with elevated levels of dopamine (Sun et al. 2022). However, the mechanisms by which probiotics could influence the activity of drugs is complex and could involve direct effects on the drug, effects on host drug transport and metabolic pathways or modification of the host gut microbiome. Moreover, individual variability in the host gut microbiome or disease associated shifts in the gut microbiome, could determine how an individual responds to a particular drug (Zhao et al. 2023). Understanding the role of the baseline host microbiome in determining probiotic responses will also be an important focus of future research (Klimenko et al. 2022; Zhao et al. 2024). In the context of psychobiotic discovery, consideration should be given to concurrent medications prescribed for mental health conditions for which psychobiotics may represent an adjunctive management strategy as well as medications prescribed for other conditions especially for psychobiotics intended for chronic use.

## 3 Other Learnings From Preclinical Models for Psychobiotic Discovery

The use of animal models offers an attractive means to explore the impact of psychobiotics and the gut microbiome on measures relevant to psychiatric conditions. Rodent models have been used in psychiatric testing for well over 100 years (Molendijk and de Kloet 2015) and there are several well characterised models to induce depression‐like phenotypes such as chronic mild stress (CMS) and behavioural screening assays such as the FST, open field test, elevated plus maze, elevated zero maze, four‐plate test and others (for detailed review of the use of animal models for assessment of anxiolytics in drug discovery see (Cryan and Sweeney 2011)). However, there can be no definitive method given the broad scope of relevant symptoms and associated behaviours, with variations in animal species, breed, sex, genetic background and method and duration of stress induction (if used) all representing important considerations among other variables. Thus, the challenges encountered in neuroscience drug discovery are now also relevant, but with a new microbial twist, to the development of efficacious psychobiotics (Markou et al. 2009). Nevertheless, many of the current clinically available antidepressants and anxiolytic drugs showed positive effects when screened using these behavioural assays, and these assays have also proven sensitive to probiotic effects (Long‐Smith et al. 2020). At the other end of the spectrum, pigs might better represent the human gastrointestinal tract given the notable differences between the human and rodent gut from structural, physiological and gut microbiota perspectives (Nguyen et al. 2015; Parois et al. 2021; Heinritz, Mosenthin, and Weiss 2013; Leser et al. 2002).

A very well‐established route to induce a depression‐like phenotype in rodent models is the CMS model (Willner 2017) developed in the early 1980s. Animals are exposed to a variety of treatments that act as ‘micro‐stressors’ such as disruption of the light/dark cycle, cage tilting, water immersion and others that are performed in an unpredictable order and at unpredictable times. As a result, the animals present with a range of neurological, physiological and behavioural signs and symptoms that are characteristic of human anxiety and depression, and which respond to antidepressants in humans. As there is no standard CMS method, the observed phenotypes are not always reproducible between labs. Nonetheless, CMS is still widely used as a model of stress‐induced depression and anxiety given the advantage offered in matching the delayed onset of action of antidepressants and anxiolytics seen in the clinic for many readouts. However, the lack of consistency has contributed to debate on reliability and reproducibility (Willner 2017). Nevertheless, in sites where reliable phenotypes are consistently produced, probiotics have been shown to reverse several or all aspects of the behavioural and molecular modifications in animals (Dandekar et al. 2022; Gao et al. 2022; Zhu et al. 2024).

The FST was originally developed as a test to screen for antidepressant drugs (Porsolt, Bertin, and Jalfre 1977). Animals are placed in a narrow water‐filled vessel and undergo a period of vigorous swimming activity, before increasingly becoming immobile and floating in place. Drugs with antidepressant effects in humans administered to rats resulted in longer active phases, tranquillisers accelerated the time to immobility, whereas anxiolytic compounds had no effect (Porsolt et al. 1978; Porsolt, Bertin, and Jalfre 1977), suggesting the FST had good predictive validity for antidepressant activity. An early assumption in FST was that immobility reflected despair, such that depression accelerated the time it took for the animal to ‘give up’ and realise that further struggle was fruitless (Porsolt, Bertin, and Jalfre 1977). However, this assumption has been questioned, and it has since been proposed that a more accurate interpretation of animal behaviour in FST is the switch from ‘active’ to ‘passive’ coping responses to an acute stressor (Molendijk and de Kloet 2015), such that immobility in FST is a coping mechanism to stress rather than a sign of despair (Commons et al. 2017). While there is ongoing debate over what immobility behaviour represents, it remains clear that this readout is responsive to conventional antidepressants, thus satisfying the requirement for predictive validity. A meta‐analysis of studies examining the effects of probiotic lactobacilli on reduction of stress, identified 10 studies and concluded that overall lactobacilli could reduce the duration of immobility by 15% (relative to controls) in stressed rodents but not in non‐stressed rodents (Mindus et al. 2021). This was observed despite significant variations between the 10 studies in terms of exactly how the FST was performed. Nonetheless, and unsurprisingly, given the difficulty of conducting meta‐analyses when there is a well acknowledged likelihood of strain‐specific effects and different biological properties, not all probiotic strains exert a benefit on the FST.

In terms of models for investigating anxiety (Cryan and Sweeney 2011), an interesting systematic review and meta‐analysis examined 25 anxiolytics shown to be effective in humans across 814 studies that employed a range of mouse behavioural tests for anxiety (Rosso et al. 2022). Of 17 outcomes (across nine common tests), five failed to show any significant effects for the anxiolytics being investigated, and only two were considered reliable in terms of detection of anxiolytic effects. Even then, the authors described significant variation in the extent, magnitude and even direction of effects, and so questioned the usefulness of current test models across drugs with different mechanisms of action. However, it is also notable that for the most commonly‐used anxiety assays for the evaluation of anxiolytic efficacy of probiotics thus far (elevated plus maze test and light–dark box), the authors noted them ‘to be sensitive in terms of detecting both a positive effect for most anxiolytic compounds and a positive effect in the majority of individual studies’ (Rosso et al. 2022). The variation in results was attributed to environmental, genetic and procedural differences and the possibility that many of the assays are more sensitive to benzodiazepines than the other different classes of antidepressants now used clinically to treat anxiety. It is also important to note that most preclinical studies have relied historically on the use of male animals and there is an urgent need to evaluate both sexes, particular in the context of translation for the currently neglected topic of women's health. Indeed, many of the observations pertaining to the gut microbiome may be sex‐dependent (Wilmes et al. 2024; Jaggar et al. 2020).

Most in vivo studies evaluating probiotics have used rodent models based on their phylogenetic proximity to humans. However, neurobiological and behavioural effects of probiotics can also be studied in alternative model systems such as the zebrafish ( *Danio rerio* ), the fruit fly ( *Drosophila melanogaster* ) and roundworm ( *Caenorhabditis elegans* ). These options also represent useful screening tools and the possibility of more rapidly accruing the mechanistic insights necessary to unravel key aspects of microbiota‐brain communication (Nagpal and Cryan 2021). For example, *B. longum* administration improved stress‐induced constipation associated with reduced cortisol levels and intestinal inflammation and restored intestinal motility and neural activity in zebrafish larvae (Lee et al. 2024). Administration of pro‐ or postbiotics also strengthens gut barrier function in zebrafish (Klewicka et al. 2015). Many of the key readouts from rodent studies are thus amenable to evaluation via these alternative model systems. It is likely that the future of microbiome research will see a much greater use of these simpler genetic model organisms as a more scalable probiotic screening solution.

In contrast to traditional psychotropic pharmaceutical compounds which are designed to produce effects via engagement with molecular targets in the CNS, the mechanism of action of psychobiotics is often unclear and requires further consideration in terms of application to animal models. For instance, probiotics act remotely from the brain and are thus considered conceptually as modifying different levels of the microbiota–gut–brain axis to achieve efficacy (Figure 2). Given that probiotics do not normally enter the bloodstream in healthy individuals, any observed effects involving microbial mediators are faced with several barriers, not limited to but including two major epithelial barriers: the gut epithelial barrier and the blood–brain barrier (Aburto and Cryan 2024). While various hypotheses have been proposed to account for the effects of psychobiotics, the exact strain‐specific mechanism(s) remain to be elucidated and could indeed differ between strains.

## 4 Experimental Strategies to Support Successful Psychobiotic Discovery

**FIGURE 3:**
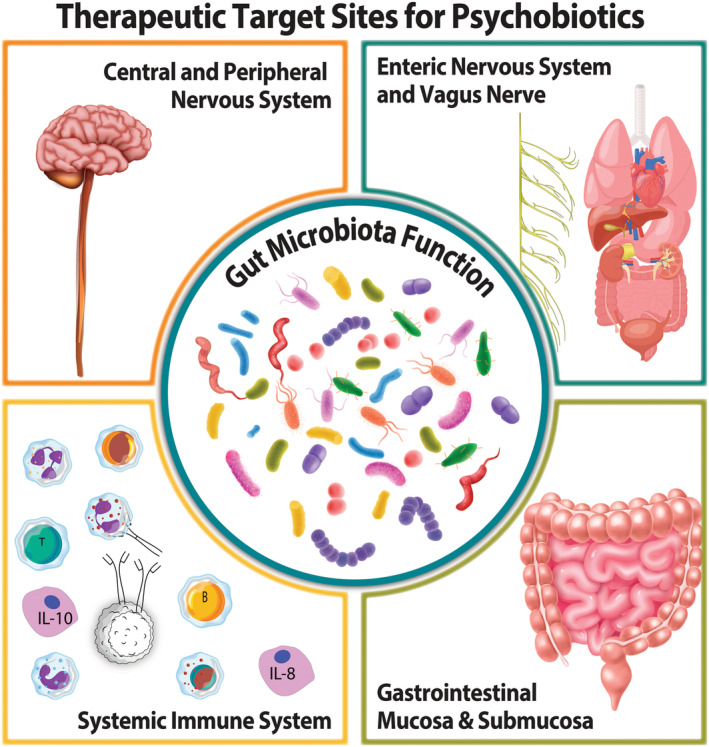
Psychobiotic administration can beneficially impact therapeutic targets locally in the gut including via the enteric nervous system and the vagus nerve, or ultimately act at distal sites to benefit systemic immune function and the CNS.

Initial bioprospecting of the gut microbiota for the selection of promising probiotic candidates includes a number of important steps based around questions such as where to sample, how to culture and how to ensure acquisition of a collection rich in promising strains (Mahmoud et al. 2022; Rocchetti et al. 2021). While these are critical steps for biodiscovery, bioprospecting is a broad term that can also be applied to the starting point for this review, which is the stage at which strains are already available for evaluation. In essence, what we are outlining here are discovery strategies to bioprospecting for psychobiotic activity. Improvements in sequencing technology and the associated availability of advanced bioinformatic pipelines provides a strong basis for in silico approaches described below. When applied at both community and strain level in conjunction with, for example, metabolomics, they have contributed to a much greater understanding of important microbial mechanisms of action that can now be evaluated using in vitro functional assays (Figures 2 and 3).

### 4.1 In Silico Approaches

#### 4.1.1 Safety Analysis

In silico safety assessments of probiotics refer to the use of computer‐based tools and algorithms to predict the safety and potential risks associated with their use. This approach is used in the early stages of probiotic discovery to identify potential hazards before proceeding to in vitro and/or in vivo testing. The process starts with genome sequencing. Whole genome sequencing (WGS) of potential probiotic strains has rapidly become the industry gold standard, not only in terms of determining taxonomy but also as a critical tool for assessing strain safety (European Food Safety 2024). WGS allows the complete DNA sequence of an organism's genome to be determined and is now required by many global regulatory authorities before new strains can be brought to market.

Illumina, Ion Torrent, DNBSEQ, Pacific Biosciences (‘PacBio’), and Oxford Nanopore Technologies (‘MinION’) represent the main next‐generation sequencing platforms of choice. Two main types of sequencing are currently used: short‐read (~250 base pairs, e.g., Illumina) and long‐read (10–100 kb, e.g., PacBio and MinION). For most probiotic strains, a combination of both short‐ and long‐read sequencing is required for good quality genome assembly that facilitates downstream bioinformatic analyses. Sequenced reads are aligned and assembled to reconstruct the complete genome sequence. Recommended methodologies for checking the quality of sequence data through to genome assembly (including software, minimum cut‐off values and other bioinformatic requirements) have recently been the subject of an EFSA consultation document (European Food Safety 2024). The assembled genome is annotated to identify the locations of genes and other functional elements. A key readout of such analyses relates to strain safety such as determining the absence of antibiotic resistance genes, virulence factors, toxins, antimicrobial compound genes and biogenic amine producing genes (Shaikh et al. 2024).

#### 4.1.2 Prediction of Psychobiotic Capabilities

More recently, advances in understanding the microbial features and properties important for host–microbe interactions suggest additional applications other than safety for WGS. Valles‐Colomer et al. (2019) described gut–brain modules (GBMs), an in silico methodology to reveal the neuroactive metabolic potential of the gut microbiota (Valles‐Colomer et al. 2019). Key metabolites that can be predicted by GBMs include GABA, serotonin, SCFAs and tryptophan. A set of 56 GBMs were assembled, each corresponding to known microbial metabolic pathways for synthesis and degradation of neurochemicals by members of the gut microbiota (Valles‐Colomer et al. 2019). The importance of GBMs for gut‐brain communication was initially demonstrated in the context of an association between microbial GABA production and depression. Specifically, the GBM analysis of faecal metagenomes identified the microbial synthesis potential of the dopamine metabolite 3,4‐dihydroxyphenylacetic acid as correlating positively with mental quality of life and indicated a potential role of microbial GABA production in alleviating depression. Subsequently, the analysis of GBMs in microbiome data has seen a wide range of applications. The functional prediction of GBMs was used to detect potential microbiome–gut–brain pathways affected by stress exposure and polyphenol treatments in animal models (Donoso et al. 2020). In rats, early‐life stress changed the abundance of bacteria linked to eight GBMs in terms of effect size, including tryptophan degradation, quinolinic acid metabolism, nitric oxide metabolism and p‐cresol synthesis compared to controls. Differentially abundant GBMs have also been identified in Alzheimer's disease, schizophrenia and anxiety/depression from re‐analysis of available sequencing datasets (Spichak et al. 2021). SCFA‐specific GBMs were upregulated in Alzheimer's disease compared to healthy controls, and potential changes in tryptophan were associated with schizophrenia and depression. This methodological concept could also be used to predict psychobiotic potential based on the genomes of individual bacterial strains. Characterisation of GBMs within a bacterial genome could predict the ability (or contribution) to produce or degrade metabolites involved in microbiome–gut–brain communication, such as GABA, serotonin, SCFAs and tryptophan. (Dalile et al. 2019; O'Riordan et al. 2022; O'Mahony et al. 2015; Donoso et al. 2023; Gheorghe et al. 2019; Roager and Licht 2018; Kaur, Bose, and Mande 2019; Lu et al. 2021). Similarly, gut metabolic modules (GMMs) can also be used to predict anaerobic fermentation capacity and gut‐specific metabolic pathways. Each GMM represents a cellular enzymatic process, defined as a set of orthologue groups, delimited by input and output metabolites (Vieira‐Silva et al. 2016). The current set of 103 GMMs captures anaerobic catabolism of carbohydrates, amino acids and lipids, cross‐feeding interactions and the production of fermentation end products, such as SCFAs. Designed as a metabolic module framework for (meta‐)genomic data analysis to study species–function relationships in gut microbial genomes and microbiomes, GMMs can also be used to predict a strain's psychobiotic potential such as production of amino acids that serve as precursors to neurotransmitters, for example, tryptophan.

### 4.2 In Vitro Functional Assessments of Microbial Mechanisms of Action

#### 4.2.1 GPCR Assays

An exciting development in understanding host–microbe interactions is the appreciation that microbial metabolites and products can interact with host receptors. Of particular interest is the recent evidence that microbiota‐derived metabolites can function as commensal ligands of GPCRs (Barki et al. 2022; Husted et al. 2017; Cryan et al. 2018; Pandey, Maharana, and Shukla 2019). GPCRs are the largest and most diverse group of cell surface receptors (Zhang et al. 2024). There are five major classes of GPCRs in humans with approximately 800–850 identified GPCRs, which accounts for ~4% of all human genes (Casado and Casado‐Anguera 2023). GPCRs play a key role in maintaining numerous aspects of host physiology through their involvement in many signalling pathways with relevance for cardiovascular, metabolic and immune health, as well as neurodevelopment and brain health.

GPCRs are ubiquitously expressed in the gut–brain axis and play a key role in neurotransmitter signalling, synaptic plasticity, blood–brain barrier regulation and neuroprotection. Thus, GPCRs may represent important targets for the metabolites produced by probiotics. Microbiota‐derived metabolites have the potential to act as ligands with orthosteric or allosteric effects to modulate the complex and dynamic downstream signalling of GPCRs (Zhang et al. 2024). Butyrate, acetate and propionate can bind the free fatty acid receptors FFAR2; GPR43 and FFAR3; GPR41, which are found in the gut lumen and dorsal root and nodose ganglia in the brain. Using chemogenic approaches it was recently shown that activation of the FFAR2 and FFAR3 can trigger sensory neuron activity at different levels in the gut–brain axis (Barki et al. 2022). In addition, bacterial structural mimicry of endogenous ligands suggests a convergence of bacterial and human signalling systems, including signalling through GPCRs (Cohen et al. 2017). For instance, a bacterial‐derived N‐acyl amide was shown to function as an agonist of the GPR119 receptor that regulates metabolic hormone secretion and glucose homeostasis (Cohen et al. 2017). An important example of this is the interaction of bacterial‐derived tryptamine, metabolised from dietary tryptophan, with the 5‐HT4 receptor in the gut (Bhattarai et al. 2018; Cryan et al. 2018). This was shown to increase intestinal fluid secretion and accelerate whole‐gut transit, which was inhibited by pharmacological blockade of the 5‐HT4 receptor and in knockout mice (Bhattarai et al. 2018).

Other recent large‐scale screening studies were able to identify several taxa‐specific microbial metabolite GPCR agonists linked to metabolism, neurotransmission and immunity (Colosimo et al. 2019). Another study using the β‐arrestin‐based PRESTO‐Tango system, identified several *Morganella morganii* strains and two *Limosilactobacillus reuteri* strains from inflammatory bowel disease (IBD) patients that were able to convert dietary histidine into histamine, with an effect on colonic motility (Chen et al. 2019).

The ghrelin receptor (growth hormone secretagogue receptor [GHSR]‐1a) is another important GPCR for gut–brain axis signalling and has been implicated in energy balance, metabolism, the central modulation of food intake, motivation, reward and mood (Lach et al. 2018; Schellekens, Dinan, and Cryan 2013; Howick et al. 2017). Studies have shown that SCFAs and other microbiota metabolites can attenuate ghrelin receptor signalling (Torres‐Fuentes et al. 2019). In one study, an in vitro cellular‐based screening approach was utilised for the characterisation of microbial metabolite–GPCR interactions based on monitoring intracellular Ca^2+^ mobilisation in cells stably transfected with the GPCR of interest (Howick et al. 2018).

These novel host–microbiota metabolome–GPCR interactions highlight the under‐resourced potential and biological relevance of the gut microbiota and its functional signalling across the gut–brain axis. Future large‐scale studies should investigate the potential of the gut microbiota metabolome to target specific GPCRs, exploring how microbial metabolites may modulate GPCR signalling through mechanisms such as antagonism, allosteric modulation, inverse agonism, functional selectivity, biased signalling and agonism.

#### 4.2.2 AhR Reporter Assays

The aryl hydrocarbon receptor (AhR) is another important receptor in the gut–brain axis, recognising multiple ligands of microbial origin (Barroso et al. 2021). AhR activity of microbial‐derived metabolites or complex community supernatants can be measured using ligand‐activated luciferase reporter assays (Gheorghe et al. 2024; Maillard et al. 2023; Rothhammer et al. 2018). Modulation of AhR activity in the gastrointestinal tract represents an important target which may be particularly important in the context of tryptophan metabolites (Meynier et al. 2022; Rueda et al. 2024). It is also notable that AhR expression on CNS cell types such as astrocytes may have important implications for neuroinflammation following activation by microbial metabolites of tryptophan (Rothhammer et al. 2016).

#### 4.2.3 Assessment of Gut and Brain Barrier Function

Barrier function is an important therapeutic target in the gut–brain axis, and includes the gut epithelial barrier, the blood–brain barrier and the blood–cerebrospinal fluid barrier (Aburto and Cryan 2024). Barrier function at these distinct sites interprets gut microbial signals to facilitate gut–brain communication. Both acute and chronic stress can disrupt barrier function and increase intestinal permeability (Gheorghe et al. 2024; Leigh et al. 2023). A variety of in vitro models and approaches are available to assess barrier function, including the evaluation of transepithelial electrical resistance (TEER) and para‐ and trans‐permeability to tracer molecules in different cell models of the gut and brain barrier, either at baseline or in the context of disruption with agents like lipopolysaccharide (LPS) (Knox et al. 2022; Maillard et al. 2023). These in vitro approaches can be complemented with information from in vivo (e.g., tight junction expression) and clinical studies (e.g., intestinal permeability surrogate markers such as LBP or sCD14) (Kelly et al. 2015).

#### 4.2.4 Organoids and Lab‐On‐A‐Chip Technologies

Organoid culture systems are important new additions to the portfolio of in vitro options for evaluation of host–microbiome interactions (Ahn et al. 2023) and intestinal organoids offer a sufficient level of complexity to generate important answers about the potential impact of microbial mediators on host physiology (Rubert et al. 2020). Lab‐on‐chip technologies may also offer a new approach in the future to study host–microbe interactions although further model development is required to combine the necessary experimental conditions with complex microbial consortia (Wheeler, Stoeger, and Owens 2024).

### 4.3 Ex Vivo Assessments of Gut and Brain Function

#### 4.3.1 Ussing Chambers

The Ussing chamber is a useful tool to bridge the gap between in vitro assays and in vivo studies, particularly in the context of gaining mechanistic insights about microbial molecular targets and often with a focus on the impact of probiotics on barrier function and fluid and electrolyte transport (Lomasney and Hyland 2013; Lomasney, Cryan, and Hyland 2014). Understanding the impact of microbes on the regulation of intestinal secretory and absorptive function has important implications for diarrhoea or constipation, and bowel habits which are frequently disrupted by stress and manifest in disorders of gut‐brain interaction. In particular, the Ussing chamber facilitates apical exposure of intestinal segments to microbes and their components or metabolites mimicking the in vivo context. Ex vivo tissue preparations also represent more complex physiological preparations than cell line models (e.g., Caco2) with epithelial, neural, immune and humoral elements all present, each of which may represent putative targets for novel psychobiotics, and which collectively influence intestinal secreto‐motor function. The primary readouts from the Ussing chamber, short‐circuit current (*I*_sc_) and TEER, reflect important physiological functions of the gastrointestinal tract namely electrogenic ion transport and gut permeability respectively, the latter of which has been widely associated with stress‐related psychiatric disorders (Kelly et al. 2015). The Ussing chamber can be applied to discovery efforts in different ways, either as a tool to determine relatively immediate effects on *I*_sc_ or TEER, and characterisation of mechanism of action, or for assessment of longer‐term effects on gastrointestinal physiology where animals are fed a particular intervention for a period. In this regard, both approaches have been employed to characterise the effects of probiotic strains (Lomasney, Cryan, and Hyland 2014). For example, different probiotic species can differentially influence enteric neural responses acutely compared to the effects on neural activity after in vivo administration, potentially because of more plastic neural changes in the gut (Lomasney, Cryan, and Hyland 2014). Pharmacological approaches can also be applied to identify intracellular signalling pathways regulated by putative probiotics, which can in turn influence the activity of epithelial ion channels and transporters (Lomasney and Hyland 2013). Given the symptoms associated with disorders of gut‐brain interaction, consideration for improving gastrointestinal function could be considered a beneficial property of putative psychobiotics.

In addition to barrier function, other immunomodulatory properties are also frequently evaluated and include the screening of activity of bacterial cells or supernatants in intestinal and peripheral blood mononuclear cells (Maillard et al. 2023). The anti‐inflammatory potential of candidate psychobiotics is an important functional property that can be deployed in the future against the low‐grade inflammation that is characteristic of many patients with stress‐related gastrointestinal and psychiatric disorders (Cruz‐Pereira et al. 2020).

#### 4.3.2 Organ Baths

Organ baths represent a versatile, simple and reproducible assay that is suitable for many organ sizes, facilitates the ability to measure concentration‐dependent changes to isometric contractions and provides data in real time (May, Evans, and Parry 2017). These can be used to evaluate the impact of bacterial strains on electrophysiological activity and propulsive motility of small and large intestine tissue preparations (Wood 2021). Lending themselves to the study of human tissues ex vivo, organ baths have been applied to characterise the anti‐diarrhoeal mechanism of action of *Escherichia coli* Nissle 1917 (Mutaflor) on gastrointestinal motility ex vivo, supernatants from which appeared to directly influence human smooth muscle preparations by an undefined metabolite (Bar et al. 2009). Organ baths have also been adapted for intraluminal delivery of bacteria to the tissue (Wang et al. 2010). For example, luminal addition of *L. reuteri* inhibited motor complexes in a concentration‐dependent manner in adapted organ baths (Wang et al. 2010). In a similar manner to Ussing chamber studies, tissues can be assessed ex vivo from animals fed probiotics or from models of gastrointestinal disease supplemented with probiotics to identify mechanism(s) underpinning potential beneficial effects on the amplitude and frequency of intestinal contractions (Metin, Altun, and Koyluoglu 2020). As with other ex vivo techniques, organ baths can be used to determine whether the biological effect elicited on gastrointestinal motility is driven by living probiotics or their cell free supernatants (Gong et al. 2017), valuable information with respect to determining how probiotics might exert their beneficial properties. As well as measuring parameters such as the frequency and amplitude of contractions, spatiotemporal maps of colonic migrating motor complexes can be generated using software to analyse video recordings of tissue responses ex vivo (Swaminathan et al. 2016), a method which has been applied to show that probiotics and other beneficial bacteria have strain and region‐specific actions on gastrointestinal motility that can be successfully discriminated using spatiotemporal mapping (Wu et al. 2013).

#### 4.3.3 Tissue Explants

Explant culture of gastrointestinal tissue offers another important approach to screen for biological activity of bacterial strains (Randall, Turton, and Foster 2011). This option has been used, for example, to evaluate the effects of SCFAs on gut, immune and oxidative responses (Fontinha et al. 2024). Stimulation of explants can also be used to study the release of hormones and other neuroactive compounds important for gut–brain axis signalling such as serotonin (Symonds et al. 2015; Peiris et al. 2022). Moreover, explants can be used to determine regional variation in modification of the immune response, for example distal versus proximal colon, by both probiotics and synbiotics (Shinde et al. 2020) or the impact of age on tissue responsiveness to microbial intervention on the tissue immune response (Vemuri et al. 2019). Some limitations of human tissue explant studies are tissue availability and viability (Aguilar‐Rojas, Olivo‐Marin, and Guillen 2020). However, human explant studies have provided insight into the mechanism by which the probiotic *E. coli* Nissle 1917 and a commensal *E. coli* modified the immune response which involved bacteria‐derived outer membrane vesicle–host interactions, with both probiotic and commensal *E. coli* having comparable effects on immune‐related genes (Fabrega et al. 2016).

#### 4.3.4 Electrophysiology

Electrophysiological measurements using, for example, multielectrode array systems are important tools to identify novel host–microbe interactions (Shaban et al. 2017). This approach has been applied to hippocampal slices from germ‐free and conventional animals and indices of synaptic plasticity inferred from the resulting electrophysiological readouts (Darch et al. 2021) which can also be applied to evaluate microbial metabolites. Electrophysiological techniques can also be applied to assess microbial signalling to intrinsic enteric neurones (Sarkar et al. 2016). For example, whole patch clamp recordings demonstrated that *L. rhamnosus* JB‐1 and *Bacteroides fragilis* significantly influenced enteric nerve function, and that these effects were recapitulated by the capsular exopolysaccharide, polysaccharide A, suggesting that this microbial component facilitates communication with the enteric nervous system (Mao et al. 2013).

## 5 Considerations for the Future Clinical Evaluation of Psychobiotics

Psychological experience and the associated behavioural correlates are complex phenomena influenced by a multitude of interacting environmental and physiological factors. This results in a surge of variables that can be well controlled in preclinical studies, but which come into play when translating animal models of psychobiotics into human trials. For instance, there is no current consensus on the precise composition and dynamics of a healthy gut microbiome in humans that can be applied across all stages of life (Liddicoat et al. 2024; Ghosh, Shanahan, and O'Toole 2022; Shanahan, Ghosh, and O'Toole 2021). The gut microbiota differs between males and females with sex differences in interactions between hormones, immune modulation of the gut–brain axis, risk of inflammation‐related disease and psychological problems (Holingue et al. 2020; Kim et al. 2023). Thus, psychobiotics may have different effects on mental wellbeing depending on host genetics. For example, Gualtieri et al. (2020) found beneficial effects of a nine‐strain combination of probiotics on reduction of anxiety symptoms in carriers of the Allele A of IL‐1*β* gene compared to non‐carriers and placebo recipients (Gualtieri et al. 2020).

Lifestyle factors further influence response to probiotics. For instance, animal models suggest that up to 50% of the composition of the gut microbiota is due to diet (Zhang et al. 2010) and alterations in diet can result in compositional changes within the human microbiota within days (Bosch et al. 2024). In a randomised trial of probiotics and health behaviours, the combination of *L. helveticus* R0052 and *B. longum* R0175 was only associated with beneficial mental health outcomes when combined with healthy lifestyle behaviours (including regular physical activity and healthy eating) (Morales‐Torres et al. 2023). These results demonstrate the interaction between lifestyle factors and probiotic supplementation and suggest that rather than single factors acting in isolation, interactions between contributors to mental wellbeing are a necessary consideration in translational research in psychobiotics.

As mentioned above, heterogeneity in psychological symptoms is considerable, and two individuals with the same clinical diagnosis of depression may share only some of the same symptoms. Variability in gut microbial composition may underpin heterogeneity in depression symptomatology (Hayes, Peters, and Foster 2020). This variability may also play a role in individual responses to psychotropic medications. For instance, it has been noted that across patients with depression, 60% experienced some degree of non‐response to first line antidepressants, while 30% experienced persistent symptoms despite treatment (Rush et al. 2006). Therefore, within a clinical trial there are likely to be sub‐groups for whom different strains of psychobiotics are more or less efficacious, a factor influencing translation of promising preclinical evidence into human trials (Nikolova et al. 2021).

Multi‐strain formulations are often favoured over single strain probiotics due in part to the potential broader spectrum of efficacy and synergistic effects, although this should not be assumed, and a precise rationale for selecting the members of such cocktails are not always clear (Goh et al. 2019; Ouwehand et al. 2018). Multi‐strain formulations are more effective as adjunct therapies for *Helicobacter pylori* eradication (McFarland 2021; McFarland et al. 2016) and possibly for the management of some irritable bowel syndrome (IBS) symptoms, although the quality of the evidence precludes any strong recommendations (Goodoory et al. 2023). For neuropsychiatric outcomes including depression and anxiety, single and multi‐strain formulations have been associated with benefits at a similar rate in humans, with single strain interventions being more effective in animal models (Joseph and Law 2019).

### 5.1 Trial Design Factors

#### 5.1.1 Psychobiotic Selection, Dose and Intervention Period

Strain specificity is known to be relevant as different strains exert different effects on the microbiome–gut–brain axis (Azad et al. 2018; Wieers et al. 2019). Certain strains can reduce depressive or anxious symptoms (Ross et al. 2024). Yet even within specific strains, studies yield differing results. A recent overview of studies investigating the dose–response relationship in probiotic intervention across a spectrum of disease areas including, diarrhoea, atopic dermatitis and blood pressure reported a lack of consensus about adequate dosage, concluding that the current data does not allow recommendations (Ouwehand 2017). This is also consistent with the view from studies in IBS, where there are also issues with the availability of information about dose–response relationships (Quigley 2015). For psychological symptoms of depression, a dose of 1 × 10^9^ colony forming units (CFU) or higher was associated with significant reductions in depressive symptoms (Musazadeh et al. 2023). Guidelines for clinical trials to provide evidence of mental health benefit of a probiotic suggest that the dose used in commercial products is the same as that used in clinical trials (Gibson et al. 2017). Dose optimisation thus remains a topic of debate in general and it is currently unclear if different doses are required for the subset of probiotics that meet the definition of psychobiotic. Current definitions of probiotics, and by extension, psychobiotics do not specify dose, only that consumption is in adequate amounts (Hill et al. 2014). It should be noted that the pharmacology of probiotics may be more complex, for example the spectrum of possible host molecular targets, than that of conventional drugs (Marteau and Shanahan 2003) with many additional factors to consider around fate and activity once ingested (Derrien and van Hylckama Vlieg 2015). The duration of the intervention requires a balance between the length of time necessary to observe clinically relevant changes in the primary outcome, alongside participant burden and adherence considerations. Specific sub‐group analysis of intervention duration in an umbrella review of probiotics for depressive symptoms identified that a duration of at least eight weeks is optimal (Musazadeh et al. 2023). An additional consideration here is that most conventional antidepressants used in the clinic have a delayed onset of action, with clinical benefits often not seen until 6–8 weeks (Marx et al. 2023). The intervention period should be determined by evaluating the timeline necessary to observe a biologically relevant impact.

The success of a clinical trial relies on participant adherence. The optimal duration from an adherence perspective is complex and depends on the convenience of the overall trial design, format of the product (capsule, powder, whole food) and the degree of flexibility in the timing of consumption. For example, an intervention product that can be consumed at any time of the day, with or without food and can even be incorporated into a meal will yield better adherence than a capsule that must be taken an hour after eating breakfast, for example. When interventions require lengthy periods, alternative methods of consumption, for example incorporating the product into a standardised food, could be considered.

#### 5.1.2 Choice of Outcome Measure

Assessment of mental health is complex due in part to the heterogeneity of presenting symptoms. The choice of screening versus diagnostic measures in a clinical trial needs to be accurately matched to the primary outcome of interest. Self‐report questionnaires are useful in capturing a range of severity across symptoms including sub‐clinical symptoms but are subject to individual interpretation, level of insight into one's experience and language and communication differences (Demyttenaere et al. 2015). Structured clinical diagnostic interviews improve the accuracy of identification of participants with a mental health disorder and are better suited to studies in patient populations but are more resource intensive.

Subtle differences between questionnaires can also influence trial results (El Dib et al. 2021). Failure to adequately capture the complete spectrum of depressive symptoms can result in inability to detect changes in sub‐groups of symptoms such as anhedonia or cognitive reactivity to sad mood (Nikolova et al. 2021). Qualitative methods can capture aspects of psychological wellbeing not detected in quantitative measures yielding different outcomes but are often neglected in randomised trial designs (Le Morvan de Sequeira et al. 2022).

#### 5.1.3 Sample and Effect Size

Decisions about the primary outcome of interest and intervention period inform the required sample size of a trial (Sorkin et al. 2020). Typically, the effect size for which a trial is powered needs to be just large enough to be statistically significant. To date, studies investigating psychobiotics on psychometric tests and inflammatory markers in MDD have had a high degree of variability in sample size with a tendency to be small (Misera et al. 2021). Recent meta‐analyses on the effects of probiotics on depressive symptoms suggest that more studies with larger sample sizes are required (Misera et al. 2021; Goh et al. 2019). Maximising recruitment and retention of participants to maintain an adequate sample size supports achieving the desired statistical power to detect group differences.

#### 5.1.4 Trial Delivery

Advances in technology have led to decentralised trial procedures which allow for some or all of the data collection to be remote. Decentralised methods reduce barriers to trial participation for those who traditionally find in‐person attendance at a clinical site difficult (Brunsdon et al. 2019; Rodriguez‐Torres, Gonzalez‐Perez, and Diaz‐Perez 2021; Rogers et al. 2022). Not all trials are suited to entirely remote delivery and the choice of in‐person, remote delivery or a hybrid approach needs to be tailored to the aims of the study (Boxall et al. 2023).

In‐person or hybrid clinical trial delivery offers the advantages of controlled conditions for data and sample collection, greater adherence and the ability to gather more fine grain data regarding confounders like diet and supplement consumption but tend to be smaller in sample size. Decentralised trial methods offer the advantage of upscaling and a larger study population, but are often limited to a more generalised cohort and less individual attention for participants. Ultimately, the choice of delivery mode depends on the target group, outcome measures and biological samples required.

## 6 Conclusions and Future Directions

Therapeutic innovations are urgently required to improve the clinical management of neuropsychiatric disorders, as well as to support gastrointestinal and psychological function in those experiencing sub‐clinical symptoms, where current conventional medications often do not perform well. Direct targeting of the gut microbiome is an appealing prospect with psychobiotics an important option in this regard, and one which can support good health and wellbeing, a key sustainability development goal. Harnessing the potential of in silico approaches can create an exciting pathway for developing safe psychobiotics with appropriate biological properties. This strategy not only helps identify and prioritise strains that are most likely to influence the microbiome–gut–brain axis and enhance mental health, but it also complements established in vitro and ex vivo methods. These methods serve to validate findings from in silico studies, uncovering potential mechanisms and additional health benefits. By employing these integrated approaches, we can mitigate the risks of future preclinical and human trials while providing insightful guidance for study design, including potential endpoints and biomarkers. The fact that the initial site of action is contained within the gut may well herald an era of interventions with superior safety profiles for CNS disorders. Nonetheless, mining the gut microbiome for new candidate psychobiotics outside of the usual suspects may prove more challenging to implement from regulatory and commercial perspectives but is a worthwhile avenue to explore. Progress in this area requires mechanistic insights and careful consideration of clinical study design and readouts to expedite the transition from bench to bedside.

## Author Contributions

**Rebecca F. Slykerman:** conceptualization, writing – review and editing, writing – original draft, supervision. **Naomi Davies:** writing – review and editing, writing – original draft. **Klara Vlckova:** writing – original draft, writing – review and editing, visualization. **Kenneth J. O'Riordan:** writing – review and editing, visualization. **Shalome A. Bassett:** conceptualization, funding acquisition, writing – original draft, writing – review and editing, supervision. **James Dekker:** writing – original draft, writing – review and editing, supervision, funding acquisition, conceptualization. **Harriët Schellekens:** conceptualization, writing – original draft, writing – review and editing, visualization, supervision. **Niall P. Hyland:** writing – original draft, writing – review and editing, conceptualization, supervision. **Gerard Clarke:** conceptualization, writing – original draft, writing – review and editing, visualization, supervision. **Elaine Patterson:** writing – original draft, writing – review and editing, visualization, supervision, conceptualization.

## Conflicts of Interest

APC Microbiome Ireland is funded by Science Foundation Ireland (SFI/12/RC/2273_P2). G.C. is also in receipt of research funding from Pharmavite, Fonterra, Kerry, Reckitt and Tate and Lyle, has received honoraria from Janssen, Probi, Boehringer Ingelheim and Apsen as an invited speaker and is or has been a paid consultant for Yakult, Heel Pharmaceuticals, Bayer Healthcare and Zentiva. H.S. has received research funding from Pharmavite, Cremo, Tate & Lyle, Pepsico, Nutricia and Fonterra. K.J.O’R has received honoraria from Sanofi Genzyme and Danone. R.F.S. and N.D. have received research funding from Fonterra. K.V., E.P., J.D. and S.A.B. are employees of Fonterra.

## Data Availability

Data sharing is not applicable to this article as no new data were created or analyzed in this study.
